# 液相色谱-串联质谱法同时测定电子烟油中的102种合成大麻素类物质

**DOI:** 10.3724/SP.J.1123.2024.03017

**Published:** 2024-10-08

**Authors:** Zhe YANG, Liwei JIANG, Siyao YANG, Yidi WU, Jianxia LYU

**Affiliations:** 1.国家毒品实验室北京分中心, 北京 100164; 1. National Narcotics Laboratory Beijing Regional Center, Beijing 100164, China; 2.中国人民公安大学, 北京 100038; 2. People’s Public Security University of China, Beijing 100038, China

**Keywords:** 液相色谱-串联质谱, 合成大麻素, 电子烟油, liquid chromatography-tandem mass spectrometry (LC-MS/MS), synthetic cannabinoids (SCs), electronic cigarette oil

## Abstract

合成大麻素是目前世界上滥用最广泛的新精神活性物质之一,其被伪装成各种形态,以电子烟油的形式贩卖是其中一种主要形式。合成大麻素结构多变,更新迅速,通常单个方法所包含的合成大麻素种类有限。本研究针对电子烟油中102种合成大麻素类物质,建立了液相色谱-三重四极杆质谱法(LC-MS/MS)同时对其进行定性定量分析的方法。实验对质谱条件和液相色谱条件进行了优化,结合外标法定量,实现了对102种合成大麻素的同时定性定量分析。样品用甲醇提取,在多反应监测(MRM)模式下,以0.1%甲酸水溶液和甲醇-乙腈(1∶1, v/v)混合溶液为流动相,采用岛津Shim-pack GIST-HP C18 AQ色谱柱(100 mm×2.1 mm, 1.9 μm)进行分离,柱温40 ℃,流速0.4 mL/min,进样量1 μL。结果表明,采用该方法,102种合成大麻素可在18 min内分离,在1~100.0 μg/L范围内线性关系良好,相关系数(*r*)≥0.9915,检出限为0.01~0.30 μg/L,定量限为0.04~0.99 μg/L,满足实际样品的分析需求。采用2、10、50 μg/L 3个水平的102种合成大麻素混合标准溶液进行精密度试验,其相对标准偏差(RSD, *n*=6)为0.3%~6.0%。以空白电子烟油为基质样品,在2、10、50 μg/L 3个加标水平下进行加标回收试验,各待测物的加标回收率为80.1%~119.8%。本方法具有准确、快速、灵敏、分离效果好等优点,适用于电子烟油中102种合成大麻素类物质的定性定量测定,可满足相关鉴定工作的要求。

## Abstract (en)

Synthetic cannabinoids (SCs), which are among the most widely abused new psychoactive substances, are much more potent and have greater efficacy than natural cannabis. SCs can be disguised in various ways and are commonly sold in the form of electronic cigarette oil. SCs belong to a large family with structures consisting of a core with substituents, linker, ring with substituents, and tail. New SCs can be developed by adding substituents, such as halogen, alkyl, and alkoxy groups, to the aromatic ring system or by changing the alkyl chain length. Since the emergence of so-called first-generation SCs, subsequent developments have led to eighth-generation indole/indazole amide-based SCs. As of July 1, 2021, the entire category of SCs was added to the list of controlled substances, but implementation requires urgent improvements in detection technologies. Typically, each method is limited to a few SCs. Owing to the vast number of chemically diverse SCs and their fast update speed, the determination and identification of various types of SCs using a single method is challenging. Therefore, rapid, sensitive, and accurate quantitative methods that includes various types of SCs must be developed to meet the demand for the qualitative and quantitative analysis of new SCs in seized electronic cigarette oil.

In this study, a liquid chromatography-tandem mass spectrometry (LC-MS/MS) method was developed for the simultaneous determination of 102 SCs in electronic cigarette oil. The mass spectrometry and liquid-phase conditions influencing SC separation and determination were optimized. Using the external standard method, 102 SCs were successfully identified in electronic cigarette oil. The samples were extracted using methanol. Target analytes were separated on a Shimadzu Shim-pack GIST-HP C18 AQ column (100 mm×2.1 mm, 1.9 μm) at a column temperature of 40 ℃. The mobile phases consisted of (A) 0.1% formic acid aqueous solution and (B) methanol-acetonitrile (1∶1, v/v). The gradient elution conditions were as follows: 0-8 min, 55%A-15%A; 8-15 min, 15%A; 15-16 min, 15%A-55%A; 16-18 min, 55%A. The flow rate was 0.4 mL/min and the injection volume was 1 μL. Operating in the multiple reaction monitoring mode, the 102 SCs were identified within 18 min. Each SC exhibited a good linear relationship in the range of 1-100.0 μg/L with a correlation coefficient (*r*)≥0.9915. The limits of detection were 0.01-0.30 μg/L and the limits of quantification were 0.04-0.99 μg/L, which meet the requirements for analyzing SCs in actual samples. Precision was determined using standard solutions with 2, 10, and 50 μg/L of the SCs. The precisions (*n*=6) were 0.3%-6.0%. The recoveries of the 102 SCs, as evaluated by spiking electronic cigarette oil at low (2 μg/mL), medium (10 μg/mL), and high (50 μg/mL) levels, were 80.1%-119.8%. Good performance was observed for the analysis of real samples. The developed method is accurate, rapid, sensitive, and effective for the determination of the 102 SCs in electronic cigarette oil, satisfying the requirements for practical qualitative and quantitative analysis.

电子烟是新型烟草制品的主流产品,自2010年以来,其市场规模和销量迅速扩张^[1]^。电子烟油由于其便于运输及吸食,已经成为合成大麻素(synthetic cannabinoids, SCs)最主要的载体,即为所谓的“上头电子烟”。近年来,在电子烟油中添加合成大麻素类物质的案例逐渐增多^[2⇓⇓⇓⇓⇓⇓-9]^。

合成大麻素是模拟大麻植物主要活性成分四氢大麻酚的化学作用而合成的一类化合物,通常具有更强大的危害性和成瘾性,能产生更为强烈的兴奋、致幻等效果^[10⇓⇓-13]^。通过对其分子进行修饰,合成大麻素更新迅速,种类不断增加,自2006年出现第一代萘甲酰吲哚类起,目前已发展至第八代吲哚/吲唑酰胺类合成大麻素^[14,15]^。目前,合成大麻素类物质被伪装成各种形态,以电子烟油的形式贩卖时,其滥用方式为将合成大麻素类物质溶于电子烟油中吸食。

为应对日益严重的合成大麻素滥用问题,2021年7月1日起,我国整类列管合成大麻素类物质。因此,在实际执法实践中,对缴获电子烟油中合成大麻素的检验鉴定需求增加,与之相关的检测技术也亟待提高。因此研究快速、灵敏、准确的合成大麻素类物质的分析方法对执法实践具有十分重要的意义。

目前,对于可疑毒品缴获物中合成大麻素的检测方法主要有液相色谱法^[16]^、气相色谱-质谱法^[17⇓⇓⇓⇓⇓⇓-24]^、液相色谱-质谱法^[25⇓-27]^、核磁共振波谱法^[23,28,29]^等。但是由于合成大麻素种类繁多、更新换代迅速,通常单个方法所包含的合成大麻素种类有限。因此,本研究拟采用液相色谱-三重四极杆质谱法(LC-MS/MS)同时检测电子烟油中102种合成大麻素类物质,涵盖发现非法滥用的大部分合成大麻素种类,一次进样即可实现102种合成大麻素的筛查和定量,能够更好地服务于实际执法工作。

## 1 实验部分

### 1.1 仪器、试剂与材料

LCMS-8060NX液相色谱-三重四极杆质谱仪(日本岛津公司),液相色谱部分配备CBM-40控制器、SIL-40C X3自动进样器和CTO-40S柱温箱,质谱部分配备电喷雾离子源(ESI)。

102种合成大麻素类物质均为上海市刑事科学技术研究院与上海原思标物科技有限公司联合研制的标准物质,纯度均≥99%,详细信息列于表1。甲醇、乙腈均为色谱纯,购自上海安谱实验科技股份有限公司;实验用水为超纯水,由Milli-Q Reference超纯水机制得。

**表1:** 102种合成大麻素的保留时间和质谱参数

No.	Synthetic cannabinoid	Retention time/min	Precursor ion (m/z)	Product ions (m/z) (Collision energies/eV)
1	5F-AMP-P7AICA	2.36	335.2	290.2^*^(-25), 270.2(-29), 214.1(-32)
2	AM-2233	2.44	459.1	98.1^*^ (-27), 112.1(-24), 362.0(-21)
3	5F-BEPIRAPIM	2.78	408.2	232.1^*^ (-25), 144.0(-43)
4	N-(1-amino-3-methyl-1-oxobutan-2-yl)-1-(4-cyanobutyl)-	2.86	342.4	226.0^*^ (-26), 145.0(-43)
	1H-indazole-3-carboxamide			
5	AM-1220	3.05	383.2	127.1^*^ (-53), 286.2(-20), 155.0(-27)
6	A-796, 260	3.65	355.2	125.2^*^ (-20), 114.2(-27)
7	JWH-193	3.76	398.9	169.0^*^ (-23), 114.0(-27), 141.0(-46)
8	N-(1-amino-3-methyl-1-oxobutan-2-yl)-1-(4-fluorobutyl)-	3.81	335.1	219.1^*^ (-24), 145.0(-41), 177.0(-35)
	1H-indazole-3-carboxamide			
9	5F-ABICA	4.24	348.2	232.1^*^ (-21), 331.2(-10), 144.0(-39)
10	5F-AB-PINACA	4.49	349.2	233.1^*^ (-24), 304.2(-16), 213.1(-32)
11	AM-1248	4.61	391.3	135.1^*^ (-28), 112.1(-30), 294.2(-29)
12	5-fluoropentyl-3-py ridinoylindole	4.65	311.3	144.0^*^ (-39), 232.0(-31), 223.0(-26)
13	AB-FUBICA	4.72	368.2	109.0^*^ (-40), 252.1(-19), 351.2(-10)
14	N-(1-amino-3-methyl-1-oxobutan-2-yl)-1-(pent-4-en-1-yl)-	4.90	328.4	212.0^*^ (-19), 144.0(-36), 158.0(-32)
	1H-indole-3-carboxamide			
15	AB-FUBINACA	4.98	369.2	109.0^*^ (-44), 324.1(-16), 253.0(-24)
16	5F-ADBICA	5.05	362.2	232.1^*^ (-23), 345.2(-11), 144.0(-41)
17	PX-2	5.24	397.2	233.1^*^ (-25), 213.0(-33), 145.0(-46)
18	5F-ADB-PINACA	5.32	363.2	318.2^*^ (-16), 177.0(-36)
19	5Cl-AB-PINACA	5.44	365.2	249.0^*^ (-25), 145.0(-43), 213.0(-33)
20	ADB-FUBICA	5.50	382.2	252.1^*^ (-22), 109.0(-42)
21	ADB-FUBIATA	5.57	396.2	109.0^*^ (-45), 238.0(-26)
22	ADB-BINACA	5.73	365.2	235.1^*^ (-25), 320.2(-15), 348.2(-10)
23	4CN-MDMB-BUTINACA	5.76	371.2	226.0^*^ (-28), 145.0(-44)
24	5F-MDMB-P7AICA	5.77	378.2	318.0^*^ (-29), 298.0(-32), 206.0(-37)
25	ADB-FUBINACA	5.78	383.2	338.2^*^ (-16), 253.1(-26)
26	ADB-BUTINACA	5.95	331.2	201.1^*^ (-27), 145.0(-41), 286.2(-15)
27	5F-CUMYL-P7AICA	5.99	368.2	174.1^*^ (-32), 230.1(-24), 348.2(-23)
28	AB-PINACA	5.99	331.2	215.1^*^ (-24), 286.2(-15), 314.2(-10)
29	5F-AMB-PICA	6.03	363.2	232.1^*^ (-19), 144.0(-38), 116.0(-54)
30	4F-MMB-BUTINACA	6.08	350.4	219.0^*^ (-24), 145.0(-39), 177.0(-33)
31	4CN-CUMYL-BUTINACA	6.08	361.2	226.1^*^ (-22), 243.1(-12), 145.0(-42)
32	ADB-4en-PINACA	6.09	343.2	213.1^*^ (-24), 298.2(-16), 171.1(-39)
33	5F-QUP7AIC	6.21	378.2	233.1^*^ (-20), 145.0(-39), 117.0(-55)
34	4F-MDMB-BUTICA	6.25	363.2	218.1^*^ (-19), 144.0(-39), 116.0(-55)
35	ADBICA	6.46	344.2	214.1^*^ (-22), 144.0(-39)
36	5F-MPP-PICA	6.53	411.2	232.1^*^ (-19), 144.0(-42)
37	CUMYL-THPINACA	6.75	378.2	243.1^*^ (-22), 260.1(-12), 119.1(-25)
38	ADB-PINACA	6.81	345.2	215.1^*^ (-26), 300.2(-15), 328.2(-10)
39	5F-MDMB-PICA	6.81	377.2	232.1^*^ (-19), 144.0(-39)
40	4F-MDMB-BUTINACA	6.86	364.2	219.1^*^ (-24), 304.2(-16), 177.0(-36)
41	5F-MN-24	6.88	375.2	232.1^*^ (-23), 144.0(-41)
42	AB-CHMINACA	6.89	357.0	241.0^*^ (-26), 312.0(-17)
43	5F-CUMYL-PICA	7.01	367.2	249.1^*^ (-16), 232.1(-28), 206.1(-25)
44	AM-694	7.07	436.0	230.9^*^ (-28), 202.9(-48), 309.2(-22)
45	AMB-FUBINACA	7.10	384.2	109.0^*^ (-44), 324.2(-16), 253.1(-23)
46	1-(4-fluorobutyl)-N-(2-phenylpropan-2-yl)-1H-indazole-	7.15	354.4	219.1^*^ (-21), 119.0(-24), 145.1(-41)
	3-carboxamide			
47	MDMB-FUBICA	7.16	397.2	252.1^*^ (-17), 109.0(-37)
48	SDB-006	7.26	321.2	214.1^*^ (-20), 188.1(-20), 278.2(-18)
49	ADB-CHMICA	7.30	370.2	240.1^*^ (-23), 353.2(-11), 144.0(-41)
50	FUB-PB-22	7.43	397.1	109.0^*^ (-35), 252.1(-14)
51	5F-EDMB-PICA	7.44	391.2	232.2^*^ (-19), 144.0(-40), 116.0(-55)
52	5F-BTP7AIC	7.70	368.2	233.1^*^ (-16), 145.0(-34)
53	5F-CUMYL-PINACA	7.70	368.2	250.1^*^ (-11), 213.1(-30)
54	AM-2201	7.78	360.1	155.1^*^ (-25), 127.1(-49), 232.1(-24)
55	JWH-030	7.87	292.1	155.0^*^ (-19), 127.0(-39)
56	JWH-201	7.98	336.2	121.0^*^ (-26), 144.0(-38), 214.0(-26)
57	JWH-015	8.03	328.2	155.0^*^ (-24), 127.1(-46)
58	AMB-CHMICA	8.09	371.2	240.1^*^ (-16), 144.0(-36), 116.0(-55)
59	RCS-4	8.15	322.2	135.0^*^ (-23), 107.0(-40), 214.1(-23)
60	FUB-JWH-018	8.16	380.1	155.0^*^ (-24), 127.1(-44), 252.1(-22)
61	5Cl-ADB	8.17	394.9	250.0^*^ (-25), 214.0(-33), 145.1(-42)
62	5F-MN-18	8.24	376.2	233.1^*^ (-17), 145.0(-38), 213.1(-28)
63	JWH-250	8.27	336.2	91.0^*^ (-41), 144.0(-34)
64	MAM-2201	8.32	374.2	169.1^*^ (-26), 141.1(-43), 232.1(-26)
65	5Cl-CUMYL-PINACA	8.43	384.8	250.0^*^ (-22), 214.0(-31), 145.0(-42)
66	JWH-073	8.48	328.2	155.0^*^ (-24), 127.0(-43), 200.1(-23)
67	CUMYL-PEGACLONE	8.62	373.2	255.1^*^ (-17), 185.1(-40), 167.0(-46)
68	JWH-251	8.64	320.2	105.0^*^ (-24), 214.0(-24), 144.0(-36)
69	5F-APINACA	8.65	384.2	135.1^*^ (-20), 107.1(-45)
70	STS-135	8.65	383.2	135.1^*^ (-31), 232.1(-24), 107.1(-44)
71	MDMB-CHMICA	8.76	385.2	240.1^*^ (-19), 144.0(-38)
72	FUB-144	8.77	350.2	109.0^*^ (-39), 125.1(-22), 252.1(-22)
73	EAM-2201	8.79	388.2	183.1^*^ (-26), 232.1(-26), 155.1(-38)
74	4F-ABUTINACA	8.98	370.2	135.1^*^ (-20), 93.1(-51), 107.1(-44)
75	N-(adamantan-1-yl)-1-(pent-4-en-1-yl)-1H-indole-3-carboxamide	9.25	363.5	135.0^*^ (-28), 107.0(-42), 93.0(-45)
76	EDMB-CHMICA	9.28	399.3	240.1^*^ (-20), 144.0(-40)
77	BIM-018	9.31	343.2	127.1^*^ (-48), 215.1(-21), 273.1(-22)
78	JWH-007	9.35	356.2	155.0^*^ (-23), 127.1(-47), 228.1(-25)
79	EDMB-PINACA	9.35	374.2	215.1^*^ (-26), 300.3(-17), 145.0(-42)
80	JWH-081	9.42	372.2	185.1^*^ (-26), 214.1(-25), 127.1(-54)
81	JWH-180	9.50	356.2	197.0^*^ (-25), 186.0(-26)
82	MN-18	9.67	358.2	215.2^*^ (-19), 145.0(-34)
83	JWH-098	9.67	386.2	185.0^*^ (-25), 157.0(-42), 228.0(-35)
84	JWH-307	9.68	386.2	127.1^*^ (-50), 155.0(-22)
85	JWH-122	9.71	356.2	169.0^*^ (-24), 141.1(-41), 214.1(-23)
86	UR-144	9.74	312.2	125.1^*^ (-22), 214.1(-24), 279.2(-26)
87	JWH-019	9.78	356.2	127.1^*^ (-47), 155.0(-26), 228.1(-24)
88	FUB-APINACA	9.88	404.2	135.1^*^ (-21), 107.1(-49)
89	APICA	9.89	365.3	135.1^*^ (-29), 214.1(-23), 107.0(-42)
90	MDMB-CHMCZCA	10.22	435.3	290.2^*^ (-21), 194.1(-44), 179.1(-50)
91	JWH-210	10.34	370.2	183.1^*^ (-26), 214.1(-25), 155.1(-37)
92	5Cl-APINACA	10.41	400.2	135.1^*^ (-23), 107.1(-48)
93	5F-AMPPPCA	10.45	424.3	135.1^*^ (-24), 107.1(-48)
94	JWH-370	10.46	382.2	155.0^*^ (-22), 127.1(-47)
95	JWH-182	11.18	384.2	197.0^*^ (-28), 141.0(-50)
96	JWH-249	11.18	384.0	214.0^*^ (-28), 144.0(-41), 169.0(-28)
97	APINACA	11.71	366.2	135.1^*^ (-22), 107.1(-42)
98	EG-018	12.32	392.2	155.0^*^ (-26), 264.1(-26), 179.1(-46)
99	CB-13	13.02	369.2	171.1^*^ (-26), 155.0(-26), 299.1(-20)
100	AMPPPCA	13.52	406.3	135.1^*^ (-24), 107.1(-48), 255.1(-29)
101	ACHMINACA	13.67	392.3	135.1^*^ (-23), 93.1(-53), 107.1(-50)
102	JWH-184	13.84	342.2	155.0^*^ (-20), 127.0(-54)

* Quantitative ion.

### 1.2 标准储备液与混合标准溶液的配制

称取各标准物质,分别用甲醇溶解后定容于10 mL容量瓶中,得到102种SCs质量浓度均为100.0 mg/L的标准储备液。量取各SCs标准储备液适量,用甲醇稀释,定容于20 mL容量瓶,配制成102种SCs质量浓度均为500 μg/L的混合标准溶液。取混合标准溶液适量,分别用甲醇和基质空白提取溶液定容,配制成质量浓度分别为100.0、50.0、20.0、10.0、5.0、2.0、1.0 μg/L的系列溶剂、基质混合标准溶液,备用。

### 1.3 样品溶液的制备

称取电子烟油样本10 mg,加入10 mL甲醇,振荡混匀后超声提取30 min,经0.22 μm有机相滤膜过滤,滤液备用。

### 1.4 仪器条件

#### 1.4.1 色谱条件

岛津Shim-pack GIST-HP C18 AQ色谱柱(100 mm×2.1 mm, 1.9 μm);流动相:A为0.1%甲酸水溶液,B为甲醇-乙腈(1∶1, v/v)混合溶液;梯度洗脱程序:0~8 min, 55%A~15%A; 8~15 min, 15%A; 15~16 min, 15%A~55%A; 16~18 min, 55%A。柱温40 ℃;流速0.4 mL/min;进样量1 μL。

#### 1.4.2 质谱条件

采用电喷雾离子源正离子模式(ESI^+^),多反应监测(MRM)模式,雾化气、干燥气和加热气均为氮气,雾化气流量为3.0 L/min,干燥气流量为10.0 L/min,加热气流量为10.0 L/min;碰撞气为氩气;脱溶剂管温度为200 ℃,接口温度为350 ℃,加热块温度为200 ℃,接口电压1 kV。其他参数见表1。

## 2 结果与讨论

### 2.1 质谱条件的优化

使用质量浓度为500 μg/L的混合标准溶液,分别在正离子模式和负离子模式下进行质谱条件优化。结果表明,102种合成大麻素在ESI^+^模式下均可产生较高强度的[M+H]^+^准分子离子峰,在此基础上选择响应值高、特征性强的2~3个子离子建立MRM通道,作为定性、定量离子。优化后的各化合物的质谱参数见表1。

### 2.2 色谱条件的优化

分别以纯水和0.1%甲酸水溶液为水相,考察了检测分析结果。以0.1%甲酸水溶液为水相时,物质的响应强度明显高于以纯水为水相时的响应强度。这是因为甲酸可以增加H^+^浓度,促进[M+H]^+^离子峰的形成^[30]^,从而促进目标物离子化。因此,最终选择0.1%甲酸水溶液为水相。

此外,分别以甲醇、乙腈和甲醇-乙腈(1∶1, v/v)混合溶液为有机相,考察了检测分析结果。结果显示,合成大麻素类物质在这3种条件下均具有较好的峰形,但是单独以甲醇或乙腈为有机相时,化合物的响应强度普遍低于以甲醇-乙腈(1∶1, v/v)混合溶液为有机相时的响应强度。在此基础上,考察以甲醇-乙腈(1∶1, v/v)混合溶液和含0.1%甲酸的甲醇-乙腈(1∶1, v/v)混合溶液为有机相时的检测分析结果。虽然在有机相中加入甲酸后,大部分物质的响应强度均有所增加,但是增加幅度有限,与不加甲酸时相比,变化并不明显。因此最终选择甲醇-乙腈(1∶1, v/v)混合溶液作为有机相。

由于不同结构的合成大麻素的极性存在较大差异^[31]^,为了保证所有物质均可以在合适的时间快速出峰,优先采用梯度洗脱的方式进行色谱分析,洗脱和平衡总时间为18 min(梯度洗脱条件见1.4.1节)。在该条件下,102种合成大麻素的保留时间为1.5~14 min,色谱峰峰形较好。

### 2.3 基质效应的评价

基质效应是指样品基质中除分析物以外的其他成分所引起的检测信号增强或减弱^[32]^,电子烟油的基质一般为丙二醇和丙三醇,有些电子烟油中还发现有*N*,2,3-三甲基-2-异丙基丁酰胺、三乙酸甘油酯和尼古丁等添加物^[33]^。采用LC-MS/MS分析时,目标物的离子化效率会受样品基质类型的影响,因此需对基质效应进行评价,并采取相应的方式降低基质效应带来的影响^[34]^。实验对基质效应进行了考察,按照基质效应=空白基质溶液中待测物的响应值/纯溶剂中待测物的响应值×100%计算基质效应。当基质效应低于80%时为基质抑制效应,当基质效应高于120%时为基质增强效应,当基质效应为80%~120%时,可以认为没有基质效应的影响^[32]^。

取空白电子烟油按照1.3节方法处理,得到空白基质溶液,配制质量浓度为2、10、50 μg/L的102种合成大麻素类物质的空白基质添加溶液;并用甲醇配制以上3种质量浓度的混合标准溶液。实验结果表明:(1)在10 μg/L和50 μg/L添加水平下,102种合成大麻素的基质效应为80.3%~119.7%,没有表现出明显的基质效应;(2)在2 μg/L添加水平下,102种合成大麻素中有84种合成大麻素的基质效应为81.9%~119.5%,没有明显的基质抑制或者基质增强作用;8种合成大麻素的基质效应为41.7%~75.5%,表现为基质抑制效应;10种合成大麻素的基质效应为125.7%~248.3%,表现为基质增强效应。各目标物的基质效应结果列于表2。

**表2:** 102种合成大麻素的检出限、定量限、基质效应、回收率和精密度

No.	Synthetic cannabinoid				LOD/ (μg/ L)		LOQ/ (μg/ L)		Matrix effects/%								Recoveries/%(n=6)										RSDs/%(n=6)				
									2 μg/L		10 μg/L		50 μg/L				2 μg/L			10 μg/L			50 μg/L				2 μg/L		10 μg/L		50 μg/L
1	5F-AMP-P7AICA				0.10		0.33		118.0		104.6		103.8					101.5		100.6		100.4					3.1		1.3		0.9
2	AM-2233				0.05		0.17		73.6		93.1		102.6					99.0		87.6		92.4					2.7		1.1		1.2
3	5F-BEPIRAPIM				0.02		0.07		94.4		98.3		99.2					96.0		90.5		100.3					2.0		0.8		0.9
4	N-(1-amino-3-methyl-1-oxobutan-2-yl)-1-(4-				0.10		0.33		84.2		85.9		97.9					95.4		80.1		105.7					2.3		1.7		1.2
	cyanobutyl)-1H-indazole-3-carboxamide																																
5	AM-1220				0.05		0.17		103.3		106.5		110.1					102.7		105.6		98.5					1.7		0.8		1.2
6	A-796, 260				0.05		0.17		100.6		100.3		103.0					96.4		94.6		101.6					1.6		1.5		0.9
7	JWH-193				0.04		0.13		105.3		94.5		97.6					94.2		86.8		98.8					0.4		0.7		0.3
8	N-(1-amino-3-methyl-1-oxobutan-2-yl)-1-(4-				0.07		0.23		106.4		103.2		107.7					101.1		100.8		105.4					2.2		1.7		0.6
	fluorobutyl)-1H-indazole-3-carboxamide																																
9	5F-ABICA				0.10		0.33		119.5		99.8		105.0					103.6		93.0		102.1					4.9		1.9		1.0
10	5F-AB-PINACA				0.09		0.30		106.7		103.6		119.7					97.6		100.2		97.6					4.3		3.2		3.1
11	AM-1248				0.20		0.66		69.9		89.4		100.3					97.6		83.6		97.6					2.5		2.0		0.5
12	5-fluoropentyl-3-py ridinoylindole				0.10		0.33		116.1		105.4		105.6					95.00		105.8		95.00					3.3		3.8		2.3
13	AB-FUBICA				0.10		0.33		138.8		118.1		116.5					113.9		119.2		113.9					5.3		3.1		1.2
14	N-(1-amino-3-methyl-1-oxobutan-2-yl)-1-				0.08		0.26		117.1		101.5		104.6					91.8		93.1		91.8					4.5		2.3		0.9
	(pent-4-en-1-yl)-1H-indole-3-carboxamide																																
15	AB-FUBINACA				0.06		0.20		172.9		116.5		118.2					106.7		119.8		106.7					3.1		3.5		1.7
16	5F-ADBICA				0.09		0.30		139.7		99.3		107.3					100.5		94.6		100.5					4.4		1.9		1.1
17	PX-2				0.08		0.26		106.5		96.0		113.7					101.7		91.2		101.7					3.4		3.4		2.5
18	5F-ADB-PINACA				0.18		0.60		152.4		104.4		104.3					89.6		99.6		89.6					3.6		3.5		1.7
19	5Cl-AB-PINACA				0.08		0.26		115.7		100.9		112.8					112.5		105.6		112.5					2.3		5.1		1.6
20	ADB-FUBICA				0.04		0.13		248.3		118.3		115.4					97.0		112.2		97.0					4.8		2.9		1.6
21	ADB-FUBIATA				0.07		0.23		117.8		115.6		107.3					83.9		109.9		83.9					4.0		2.3		2.2
22	ADB-BINACA				0.08		0.26		133.5		109.5		115.9					110.0		110.4		110.0					5.1		5.2		2.8
23	4CN-MDMB-BUTINACA				0.06		0.20		113.1		98.9		116.4					99.2		97.1		99.2					2.3		3.2		2.2
24	5F-MDMB-P7AICA				0.09		0.30		110.3		104.2		104.5					100.1		96.6		100.1					5.5		3.4		1.9
25	ADB-FUBINACA				0.20		0.66		115.6		118.9		111.0					119.7		112.3		119.7					5.8		3.9		2.4
26	ADB-BUTINACA				0.09		0.30		170.4		100.9		110.9					107.3		97.6		107.3					3.9		2.5		2.5
27	5F-CUMYL-P7AICA				0.20		0.66		108.1		100.9		107.3					103.7		101.8		103.7					5.4		1.7		1.7
28	AB-PINACA				0.10		0.33		125.7		102.9		106.6					105.4		95.8		105.4					5.1		2.5		0.6
29	5F-AMB-PICA				0.02		0.07		109.3		100.0		106.5					97.7		96.5		97.7					2.2		1.9		1.7
30	4F-MMB-BUTINACA				0.10		0.33		90.8		92.9		97.7					95.1		85.3		95.1					3.2		3.3		3.3
31	4CN-CUMYL-BUTINACA				0.03		0.10		89.3		91.9		104.4					102.6		93.2		102.6					4.7		3.3		1.5
32	ADB-4en-PINACA				0.10		0.33		117.6		102.2		110.9					97.3		99.8		97.3					5.9		2.9		1.4
33	5F-QUP7AIC				0.03		0.10		60.8		87.7		97.6					98.6		80.6		98.6					3.6		2.8		2.3
34	4F-MDMB-BUTICA				0.02		0.07		95.1		103.2		104.9					101.4		99.6		101.4					5.5		1.9		2.3
35	ADBICA				0.04		0.13		150.3		114.6		110.0					104.6		111.6		104.6					4.3		3.3		1.8
36	5F-MPP-PICA				0.02		0.07		108.9		103.6		112.9					104.8		98.6		104.8					2.9		2.7		4.0
37	CUMYL-THPINACA				0.05		0.17		93.9		90.8		98.8					91.8		81.4		91.8					4.9		5.5		2.2
38	ADB-PINACA				0.30		0.99		116.9		86.8		112.9					117.6		86.1		117.6					5.0		2.7		2.8
39	5F-MDMB-PICA				0.01		0.04		101.8		101.3		110.4					94.8		96.7		94.8					4.8		1.4		3.8
40	4F-MDMB-BUTINACA				0.09		0.30		95.9		97.3		101.9					98.9		92.2		98.9					5.9		2.4		2.5
41	5F-MN-24				0.02		0.07		106.6		112.7		115.4					100.2		109.3		100.2					4.2		5.6		0.9
42	AB-CHMINACA				0.05		0.17		91.6		103.9		108.8					100.3		94.7		100.3					6.0		4.9		2.9
43	5F-CUMYL-PICA				0.02		0.07		91.4		96.8		105.2					93.0		86.1		93.0					0.9		3.2		1.9
44	AM-694				0.05		0.17		91.3		97.1		103.3					94.0		96.6		94.0					4.6		2.7		2.4
45	AMB-FUBINACA				0.05		0.17		98.4		91.9		111.8					93.9		88.9		93.9					4.8		2.5		2.4
46	1-(4-fluorobutyl)-N-(2-phenylpropan-2-yl)-				0.03		0.10		88.1		89.2		101.7					98.0		82.1		98.0					3.5		2.6		3.6
	1H-indazole-3-carboxamide																																
47	MDMB-FUBICA				0.02		0.07		65.1		94.6		110.0					102.7		88.8		102.7					2.6		2.2		1.2
48	SDB-006				0.10		0.33		85.9		103.2		102.2					103.0		94.8		103.0					3.0		2.9		2.0
49	ADB-CHMICA				0.10		0.33		105.8		110.4		104.4					87.5		100.7		87.5					5.7		3.8		0.9
50	FUB-PB-22				0.02		0.07		97.0		99.2		107.7					116.9		97.1		116.9					3.7		2.1		2.1
51	5F-EDMB-PICA				0.02		0.07		83.4		92.4		102.5					98.9		86.9		98.9					2.9		3.9		1.1
52	5F-BTP7AIC				0.05		0.17		88.4		83.5		97.4					86.6		80.8		86.6					4.9		2.6		2.4
53	5F-CUMYL-PINACA				0.30		0.99		96.8		95.5		105.5					96.9		89.9		96.9					4.8		3.2		1.9
54	AM-2201				0.01		0.04		88.0		92.3		105.4					101.0		88.5		101.0					3.6		1.9		2.3
55	JWH-030				0.02		0.07		101.8		93.6		102.7					95.1		89.2		95.1					2.8		3.6		1.8
56	JWH-201				0.02		0.05		93.2		97.2		111.5					97.4		92.7		110.5					2.7		3.6		2.6
57	JWH-015				0.02		0.07		105.3		91.5		103.2					100.9		86.6		105.6					2.8		3.3		4.6
58	AMB-CHMICA				0.02		0.07		94.8		92.3		99.5					85.4		86.8		98.5					4.9		4.3		3.9
59	RCS-4				0.07		0.23		88.1		99.8		117.6					100.3		95.1		118.9					2.1		1.6		2.3
60	FUB-JWH-018				0.06		0.20		104.9		117.8		115.2					100.3		118.1		113.3					4.6		5.2		2.3
61	5Cl-ADB				0.29		0.97		81.9		95.5		104.8					87.4		84.1		104.1					4.2		5.1		3.1
62	5F-MN-18				0.05		0.17		71.1		80.3		102.2					90.9		81.6		94.9					2.4		3.7		3.3
63	JWH-250				0.28		0.92		87.0		98.4		107.9					100.3		91.2		104.9					1.9		3.8		2.6
64	MAM-2201				0.03		0.10		86.2		90.4		95.1					99.9		87.0		116.8					5.6		1.8		1.8
65	5Cl-CUMYL-PINACA				0.19		0.63		88.5		83.8		102.7					96.8		80.9		103.9					5.6		5.8		3.1
66	JWH-073				0.03		0.10		100.9		102.6		102.5					97.9		101.7		102.7					3.6		4.4		3.2
67	CUMYL-PEGACLONE				0.01		0.04		91.6		95.1		106.4					94.6		85.7		100.6					3.0		3.5		3.2
68	JWH-251				0.06		0.20		83.0		97.4		108.1					92.2		90.7		105.1					3.7		2.5		2.1
69	5F-APINACA				0.06		0.20		109.5		83.7		90.8					87.5		82.8		91.0					5.2		5.9		5.4
70	STS-135				0.01		0.04		110.2		103.6		113.5					105.3		106.9		79.4					4.4		2.9		3.4
71	MDMB-CHMICA				0.01		0.04		105.1		99.2		108.3					98.8		97.1		109.1					1.4		1.1		1.9
72	FUB-144				0.05		0.17		97.0		98.8		106.9					100.2		92.5		104.9					5.8		4.6		2.9
73	EAM-2201				0.02		0.07		85.7		83.8		102.2					84.6		80.3		107.4					2.9		4.3		4.6
74	4F-ABUTINACA				0.02		0.06		92.1		84.7		103.2					102.1		80.4		105.4					3.7		2.2		3.8
75	N-(adamantan-1-yl)-1-(pent-4-en-1-yl)-				0.06		0.20		98.4		80.5		94.4					80.1		85.2		90.6					5.4		3.9		1.9
	1H-indole-3-carboxamide																																
76	EDMB-CHMICA				0.05		0.17		94.4		92.4		107.9					97.4		88.6		91.9					2.7		2.1		1.8
77	BIM-018				0.02		0.07		105.9		100.0		104.4					99.1		96.5		104.6					2.4		3.4		3.7
78	JWH-007				0.02		0.07		83.5		92.7		102.1					93.9		89.6		100.9					1.1		1.6		2.9
79	EDMB-PINACA				0.02		0.07		89.9		102.8		109.6					100.4		103.6		111.5					3.9		2.8		2.4
80	JWH-081				0.02		0.07		102.2		92.3		107.7					97.9		91.3		108.4					3.9		1.7		3.7
81	JWH-180				0.02		0.07		90.9		85.6		100.2					94.7		83.3		99.9					4.5		3.7		3.5
82	MN-18				0.06		0.20		85.5		86.9		99.5					92.5		83.1		97.9					3.3		2.4		3.9
83	JWH-098				0.01		0.04		116.6		102.3		96.6					100.3		99.5		101.4					2.2		1.1		2.1
84	JWH-307				0.03		0.10		94.5		95.0		93.7					99.2		93.2		108.9					3.0		1.0		1.3
85	JWH-122				0.02		0.07		89.1		90.7		93.6					95.8		86.7		92.1					3.7		1.4		2.9
86	UR-144				0.07		0.23		85.9		90.1		91.7					84.9		85.8		89.0					4.4		1.1		2.0
87	JWH-019				0.02		0.07		87.2		90.9		106.4					95.2		90.5		105.2					2.2		1.1		5.6
88	FUB-APINACA				0.06		0.20		99.0		81.5		80.5					96.9		81.5		99.2					2.9		1.4		3.6
89	APICA				0.02		0.07		113.6		81.6		82.4					94.8		80.5		82.0					3.6		1.3		2.3
90	MDMB-CHMCZCA				0.03		0.10		41.7		85.4		101.5					102.7		81.4		94.3					2.8		0.8		1.1
91	JWH-210				0.01		0.04		91.0		97.9		107.6					98.1		97.8		108.3					2.4		1.1		3.6
92	5Cl-APINACA				0.04		0.13		75.5		88.1		103.6					98.4		85.9		103.0					1.9		2.9		2.5
93	5F-AMPPPCA				0.04		0.13		102.2		95.2		108.8					99.2		96.1		110.8					0.8		1.1		2.4
94	JWH-370				0.01		0.04		103.5		98.2		104.9					98.7		97.8		96.5					1.9		0.9		2.3
95	JWH-182				0.02		0.07		100.0		95.9		100.8					94.6		92.1		97.9					1.3		0.9		2.3
96	JWH-249				0.10		0.33		93.3		97.6		100.3					95.5		93.9		91.6					2.1		1.6		1.6
97	APINACA				0.05		0.17		61.9		83.2		99.6					96.4		81.7		97.1					0.9		1.9		2.1
98	EG-018				0.20		0.66		109.3		93.6		107.8					96.1		91.2		105.5					0.8		1.9		1.5
99	CB-13				0.20		0.66		97.9		96.9		110.7					100.2		94.0		109.6					1.1		1.0		1.2
100	AMPPPCA				0.30		0.99		108.8		94.2		110.6					100.0		95.6		109.9					0.4		1.5		3.8
101	ACHMINACA				0.10		0.33		85.4		86.6		105.2					96.9		84.9		102.1					0.8		1.2		3.2
102	JWH-184				0.20		0.66		128.9		88.3		96.5					92.9		80.9		97.3					2.3		0.5		1.2

The linear range was 1-100.0 μg/L, and the correlation coefficients (*r*) were 0.9915-0.9998.

在2 μg/L添加水平下部分合成大麻素表现出基质抑制或基质增强效应,这可能与目标物的极性以及浓度较低有关。目前常用的减少基质效应影响的方法有同位素内标、减少进样量、基质匹配标准曲线等,其中基质匹配标准曲线法效果良好,且操作简单、经济实用^[34,35]^,适合本实验使用。因此,为了使定量更加准确、可靠,本实验采用基质匹配标准曲线的方法进行定量。

### 2.4 方法学评价

#### 2.4.1 线性关系、检出限与定量限

向不含分析物的空白电子烟油提取液中添加102种合成大麻素的混合标准溶液,配制成不同质量浓度的系列混合标准溶液进行测定,以各组分的质量浓度(*x*, μg/L)为横坐标,峰面积(*y*)为纵坐标绘制曲线,曲线采用线性拟合。结果显示,102种合成大麻素在1~100.0 μg/L范围内线性关系良好,相关系数*r*为0.9915~0.9998。分别以信噪比(*S/N*)=3和*S/N*=10确定检出限(LOD)和定量限(LOQ)^[36⇓-38]^。结果显示,102种合成大麻素的检出限为0.01~0.30 μg/L,定量限为0.04~0.99 μg/L,表示该方法具有较高的灵敏度,可以满足电子烟油中合成大麻素的日常检测需求。线性相关系数、检出限和定量限见表2。

#### 2.4.2 回收率

向不含分析物的空白电子烟油样品中添加102种合成大麻素的混合标准溶液,在低(2 μg/L)、中(10 μg/L)、高(50 μg/L)3个水平下进行加标回收试验,每个加标水平平行测定6次,计算回收率。计算得出,各分析物的加标回收率为80.1%~119.8%,相对标准偏差(RSD, *n*=6)为0.7%~7.1%,表明该方法的定量分析结果具有准确性和可靠性。102种合成大麻素的加标回收率见表2。

#### 2.4.3 精密度

制备质量浓度分别为2、10、50 μg/L的102种合成大麻素混合标准溶液,每个质量浓度平行测定6次,计算得到精密度(RSD),相关数据见表2。由表2可知,102种合成大麻素的日内精密度为0.3%~6.0%。由结果可知,该方法的精密度良好。

### 2.5 方法对比

将本文中所建立的方法与其他文章中电子烟油中合成大麻素的检测方法进行对比,如表3所示。本方法可同时检测电子烟油中102种合成大麻素,检测种类包括大部分合成大麻素类物质,且分析时间较短,检测效率较高。该方法的检出限和定量限处于较低水平,能够满足日常检测的需求。

**表3:** 本方法与其他方法的比较

Actual sample	Number of target compounds	Detection method	Instrumental analysis time/min	LOD	LOQ	Ref.
Electronic cigarette oil	102	LC-MS/MS	18	0.01-0.3 μg/L	0.033-0.99 μg/L	this study
Electronic cigarette oil, urine	1	GC-MS	17	-	-	[5]
Electronic cigarette oil	5	UPLC	10	0.2 mg/L	0.6 mg/L	[16]
Electronic cigarette oil	9	GC-MS	20	0.04-0.25 mg/L	0.15-0.85 mg/L	[19]
Electronic cigarette oil	58	HPLC-MS/MS	12	0.5-1 μg/g	2 μg/g	[25]
Electronic cigarette oil	5	GC-MS	17	-	0.025 mg/mL	[33]

-: no data.

### 2.6 实际样品检测

利用该方法对案件中缴获的7份电子烟油样品进行分析。按1.3节方法对样品进行预处理后,再根据1.4节的仪器条件对样品进行分析。在7份电子烟油样品中,检出4F-MDMB-BUTICA、4CN-CUMYL-BUTINACA、4F-ABUTINACA、ADB-BUTINACA、5F-MDMB-PICA、4F-MDMB-BUTINACA和ADB-4en-PINACA共7种合成大麻素,含量为0.023~6.90 μg/mg,其提取离子色谱图见图1,每份样品中合成大麻素的详细含量见表4。

**图1:**
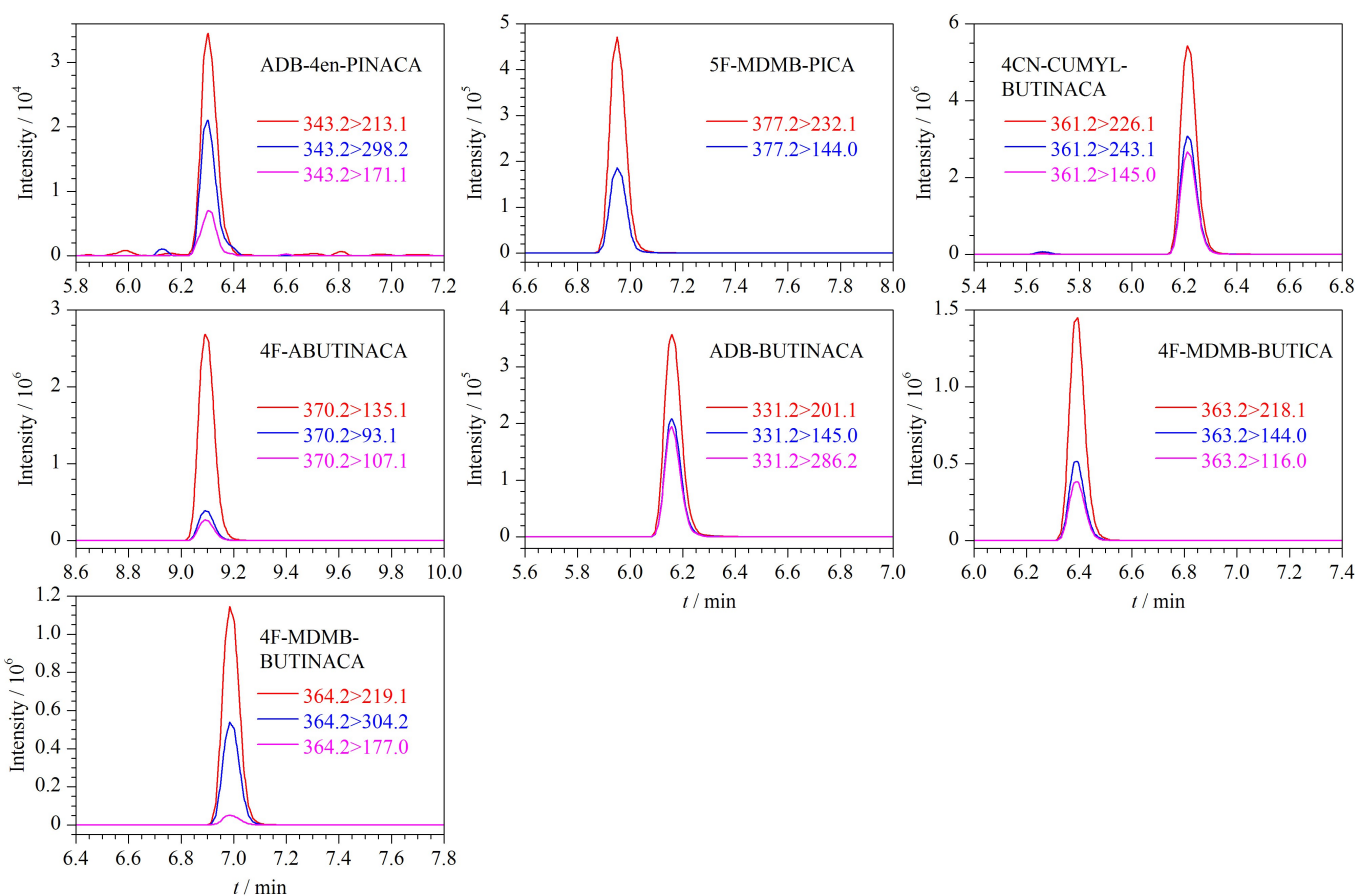
实际电子烟油样品中7种合成大麻素的提取离子色谱图

**表4:** 实际电子烟油样品中合成大麻素的检测结果

Sample	Contents of synthetic cannabinoids/(μg/mg)						
	4F-MDMB- BUTICA	4CN-CUMYL- BUTINACA	4F- ABUTINACA	ADB- BUTINACA	5F-MDMB- PICA	4F-MDMB- BUTINACA	ADB-4en- PINACA
1	-	2.48	-	-	0.023	-	0.059
2	-	6.90	-	-	-	-	0.049
3	-	-	1.59	0.58	-	-	-
4	0.84	-	1.25	0.14	-	-	-
5	-	-	2.46	0.031	-	-	-
6	-	-	-	-	1.12	0.71	-
7	-	-	-	-	0.21	-	-

-: not detected

合成大麻素更新迅速,种类不断增加,本方法包含102种合成大麻素类物质,包括国内已发现的大部分合成大麻素类物质,具有较高的实用性。

## 3 结论

本文建立了液相色谱-三重四极杆质谱同时检测电子烟油中102种合成大麻素类物质的方法,该法可在18 min内同时对102种合成大麻素进行定性和定量分析。经过实际样品验证,本方法具有准确、快速、灵敏等优点,适用于电子烟油中102种合成大麻素成分的定性和定量分析,能够满足相关鉴定工作的要求,具有较高的实用性,可以为相关案件中合成大麻素的鉴定提供参考。
